# Estimates, trends, and drivers of the global burden of type 2 diabetes attributable to PM_2·5_ air pollution, 1990–2019: an analysis of data from the Global Burden of Disease Study 2019

**DOI:** 10.1016/S2542-5196(22)00122-X

**Published:** 2022-07-06

**Authors:** GBD 2019 Diabetes and Air Pollution Collaborators, Katrin Burkart, Kate Causey, Aaron J Cohen, Sarah S Wozniak, Devashri Digvijay Salvi, Cristiana Abbafati, Victor Adekanmbi, Jose C Adsuar, Keivan Ahmadi, Fares Alahdab, Ziyad Al-Aly, Vahid Alipour, Nelson Alvis-Guzman, Adeladza Kofi Amegah, Catalina Liliana Andrei, Tudorel Andrei, Fereshteh Ansari, Jalal Arabloo, Olatunde Aremu, Timur Aripov, Ebrahim Babaee, Maciej Banach, Anthony Barnett, Till Winfried Bärnighausen, Neeraj Bedi, Masoud Behzadifar, Yannick Béjot, Derrick A Bennett, Isabela M Bensenor, Robert S Bernstein, Krittika Bhattacharyya, Ali Bijani, Antonio Biondi, Somayeh Bohlouli, Susanne Breitner, Hermann Brenner, Zahid A Butt, Luis Alberto Cámera, Carlos Cantu-Brito, Felix Carvalho, Ester Cerin, Vijay Kumar Chattu, Bal Govind Chauhan, Jee-Young Jasmine Choi, Dinh-Toi Chu, Xiaochen Dai, Lalit Dandona, Rakhi Dandona, Ahmad Daryani, Kairat Davletov, Barbora de Courten, Feleke Mekonnen Demeke, Edgar Denova-Gutiérrez, Samath Dhamminda Dharmaratne, Meghnath Dhimal, Daniel Diaz, Shirin Djalalinia, Bruce B Duncan, Maysaa El Sayed Zaki, Sharareh Eskandarieh, Mohammad Fareed, Farshad Farzadfar, Nazir Fattahi, Mehdi Fazlzadeh, Eduarda Fernandes, Irina Filip, Florian Fischer, Nataliya A Foigt, Marisa Freitas, Ahmad Ghashghaee, Paramjit Singh Gill, Ibrahim Abdelmageed Ginawi, Sameer Vali Gopalani, Yuming Guo, Rajat Das Gupta, Tesfa Dejenie Habtewold, Randah R Hamadeh, Samer Hamidi, Graeme J Hankey, Edris Hasanpoor, Hamid Yimam Hassen, Simon I Hay, Behzad Heibati, Michael K Hole, Naznin Hossain, Mowafa Househ, Seyed Sina Naghibi Irvani, Jalil Jaafari, Mihajlo Jakovljevic, Ravi Prakash Jha, Jost B Jonas, Jacek Jerzy Jozwiak, Amir Kasaeian, Neda Kaydi, Yousef Saleh Khader, Morteza Abdullatif Khafaie, Ejaz Ahmad Khan, Junaid Khan, Md Nuruzzaman Khan, Khaled Khatab, Amir M Khater, Yun Jin Kim, Ruth W Kimokoti, Adnan Kisa, Mika Kivimäki, Luke D Knibbs, Soewarta Kosen, Parvaiz A Koul, Ai Koyanagi, Barthelemy Kuate Defo, Nuworza Kugbey, Paolo Lauriola, Paul H Lee, Mostafa Leili, Sonia Lewycka, Shanshan Li, Lee-Ling Lim, Shai Linn, Yong Liu, Stefan Lorkowski, Phetole Walter Mahasha, Narayan B Mahotra, Azeem Majeed, Afshin Maleki, Reza Malekzadeh, Abdullah A Mamun, Navid Manafi, Santi Martini, Birhanu Geta Meharie, Ritesh G Menezes, Tomislav Mestrovic, Bartosz Miazgowski, Tomasz Miazgowski, Ted R Miller, GK Mini, Andreea Mirica, Erkin M Mirrakhimov, Bahram Mohajer, Shafiu Mohammed, Viswanathan Mohan, Ali H Mokdad, Lorenzo Monasta, Paula Moraga, Shane Douglas Morrison, Ulrich Otto Mueller, Satinath Mukhopadhyay, Ghulam Mustafa, Saravanan Muthupandian, Gurudatta Naik, Vinay Nangia, Duduzile Edith Ndwandwe, Ruxandra Irina Negoi, Dina Nur Anggraini Ningrum, Jean Jacques Noubiap, Felix Akpojene Ogbo, Andrew T Olagunju, Obinna E Onwujekwe, Alberto Ortiz, Mayowa O Owolabi, Mahesh P A, Songhomitra Panda-Jonas, Eun-Kee Park, Fatemeh Pashazadeh Kan, Meghdad Pirsaheb, Maarten J Postma, Hadi Pourjafar, Amir Radfar, Alireza Rafiei, Fakher Rahim, Vafa Rahimi-Movaghar, Muhammad Aziz Rahman, Rajesh Kumar Rai, Chhabi Lal Ranabhat, Samira Raoofi, Lal Rawal, Andre M N Renzaho, Aziz Rezapour, Daniela Ribeiro, Leonardo Roever, Luca Ronfani, Siamak Sabour, Basema Saddik, Ehsan Sadeghi, Sahar Saeedi Moghaddam, Amirhossein Sahebkar, Mohammad Ali Sahraian, Hamideh Salimzadeh, Sundeep Santosh Salvi, Abdallah M Samy, Juan Sanabria, Rodrigo Sarmiento-Suárez, Thirunavukkarasu Sathish, Maria Inês Schmidt, Aletta Elisabeth Schutte, Sadaf G Sepanlou, Masood Ali Shaikh, Kiomars Sharafi, Aziz Sheikh, Mika Shigematsu, Rahman Shiri, Reza Shirkoohi, Kerem Shuval, Ireneous N Soyiri, Rafael Tabarés-Seisdedos, Yonatal Mesfin Tefera, Arash Tehrani-Banihashemi, Mohamad-Hani Temsah, Kavumpurathu Raman Thankappan, Roman Topor-Madry, Lorainne Tudor Car, Irfan Ullah, Marco Vacante, Pascual R Valdez, Tommi Juhani Vasankari, Francesco S Violante, Yasir Waheed, Charles D A Wolfe, Tomohide Yamada, Naohiro Yonemoto, Chuanhua Yu, Sojib Bin Zaman, Yunquan Zhang, Sanjay Zodpey, Stephen S Lim, Jeffrey D Stanaway, Michael Brauer

## Abstract

**Background:**

Experimental and epidemiological studies indicate an association between exposure to particulate matter (PM) air pollution and increased risk of type 2 diabetes. In view of the high and increasing prevalence of diabetes, we aimed to quantify the burden of type 2 diabetes attributable to PM_2·5_ originating from ambient and household air pollution.

**Methods:**

We systematically compiled all relevant cohort and case-control studies assessing the effect of exposure to household and ambient fine particulate matter (PM_2·5_) air pollution on type 2 diabetes incidence and mortality. We derived an exposure–response curve from the extracted relative risk estimates using the MR-BRT (meta-regression—Bayesian, regularised, trimmed) tool. The estimated curve was linked to ambient and household PM_2·5_ exposures from the Global Burden of Diseases, Injuries, and Risk Factors Study 2019, and estimates of the attributable burden (population attributable fractions and rates per 100 000 population of deaths and disability-adjusted life-years) for 204 countries from 1990 to 2019 were calculated. We also assessed the role of changes in exposure, population size, age, and type 2 diabetes incidence in the observed trend in PM_2·5_-attributable type 2 diabetes burden. All estimates are presented with 95% uncertainty intervals.

**Findings:**

In 2019, approximately a fifth of the global burden of type 2 diabetes was attributable to PM_2·5_ exposure, with an estimated 3·78 (95% uncertainty interval 2·68–4·83) deaths per 100 000 population and 167 (117–223) disability-adjusted life-years (DALYs) per 100 000 population. Approximately 13·4% (9·49–17·5) of deaths and 13·6% (9·73–17·9) of DALYs due to type 2 diabetes were contributed by ambient PM2·5, and 6·50% (4·22–9·53) of deaths and 5·92% (3·81–8·64) of DALYs by household air pollution. High burdens, in terms of numbers as well as rates, were estimated in Asia, sub-Saharan Africa, and South America. Since 1990, the attributable burden has increased by 50%, driven largely by population growth and ageing. Globally, the impact of reductions in household air pollution was largely offset by increased ambient PM_2·5_.

**Interpretation:**

Air pollution is a major risk factor for diabetes. We estimated that about a fifth of the global burden of type 2 diabetes is attributable PM_2·5_ pollution. Air pollution mitigation therefore might have an essential role in reducing the global disease burden resulting from type 2 diabetes.

**Funding:**

Bill & Melinda Gates Foundation.

## Introduction

Diabetes has been highlighted as a major global health threat by WHO.^1^ The disease is characterised by hyperglycaemia resulting from dysfunctional insulin secretion or action.^2^ Long-term consequences can be dysfunction and failure of different organs, especially the eyes, kidneys, nerves, heart, and blood vessels.^2^ Type 2 diabetes accounts for 90–99% of diabetes cases globally and contributed to approximately 94% of disability-adjusted life-years (DALYs) and 96% of years lived with disability (YLDs) due to diabetes.^2^, ^3^, ^4^

In total, the global burden of diabetes was estimated at 1·55 million excess deaths, with 34 million years of life lost (YLLs) and 37 million YLDs in 2019.^3^ Since the 1990s, global age-standardised death rates increased by 8·6%, all-age death rates increased by 62%, and the burden of disease has approximately doubled.^3^ Recent projections predict a further increase in diabetes-related mortality rates of approximately 75% by 2040.^5^ Metabolic and behavioural risk factors, such as obesity, smoking, diet, and physical inactivity, have been highlighted as major contributors to the burden of type 2 diabetes.^1^ In the past 15 years, studies have indicated an important role of factors that promote inflammatory responses,^4^, ^6^ such as air pollution. A meta-analysis that included five cross-sectional and five prospective cohort studies showed an increased risk of type 2 diabetes due to exposure to particulate matter (PM) or nitrogen dioxide air pollution.^7^

**Research in context**

**Evidence before this study**

Type 2 diabetes is a major global health concern, contributing to approximately 1·5 million deaths, 31 million years of life lost, and 35 million years lived with disability in 2019. Behavioural and metabolic risk factors are widely acknowledged to increase diabetes incidence, but epidemiological and experimental evidence also supports a relationship between long-term exposure to fine particulate matter (PM_2·5_) air pollution and increased diabetes incidence and mortality. The well documented increase in incidence and mortality from inflammation has been highlighted as a relevant biological mechanism linking air pollution exposure to diabetes. Although the effect of exposure to ambient and household PM_2·5_ pollution on diabetes has been estimated in different locations and population groups, the current global disease burden attributable to PM_2·5_, its temporal trends, and its magnitude relative to other known diabetes risk factors have not yet been evaluated. The framework of the Global Burden of Diseases, Injuries, and Risk Factors Study (GBD) allows a systematic assessment of the contribution of individual risk factors in a spatially and temporally explicit manner, producing comparable estimates at global, regional, and country levels.

**Added value of this study**

We systematically reviewed existing epidemiological studies on the relationship between PM_2·5_ exposure and type 2 diabetes from diverse sources. We created an exposure–response relationship using the meta-regression tool MR-BRT (meta-regression—Bayesian, regularised, trimmed). The regularisation feature provided by the MR-BRT tool allowed us to fit a spline covering the entire global exposure range by putting a strong prior on the shape of the curve at the upper end of the exposure range and keeping it from increasing. By linking the derived exposure–response curve to modelled ambient and household air pollution levels, we estimated the population attributable fraction (PAF) and derived the type 2 diabetes burden attributable to PM_2·5_ air pollution for 204 countries and territories from 1990 to 2019. Using a proportional PAF approach, we estimated the relative contribution of ambient and household air pollution. To better understand the drivers behind the observed trend in PM_2·5_-attributable diabetes burden, we assessed the role of changes in exposure, population size, age, and diabetes incidence. Approximately a fifth of the global diabetes burden in 2019 was related to exposure to PM_2·5_ air pollution. Since 1990, the attributable burden has doubled, driven largely by population growth and ageing. Reductions in household air pollution burden were largely offset by increased burden from exposure to ambient PM_2·5_. Around 80% of the attributable burden occurred in Asia, sub-Saharan Africa, and South America.

**Implications of all the available evidence**

Our results highlight the importance of PM_2·5_ air pollution as a risk factor for diabetes and as target for risk reduction. Most of the PM_2·5_-attributable burden stems from ambient air pollution, except in sub-Saharan Africa, where household air pollution was the major contributor. Although increasing ambient air pollution levels in low-income and middle-income countries contributed to the rise in the diabetes burden attributable to air pollution observed between 1990 and 2019, population growth and ageing were the major drivers of this trend, and predicted demographic trends suggest further increases in the diabetes burden associated with PM_2·5_ air pollution in the future.

Given the relationship between exposure to air pollution and cardiovascular disease^8^ and similarities in biological pathways between diabetes and cardiovascular disease, air pollution has been posited to have a causal role in diabetes.^9^ Experimental studies indicate the role of inflammation in biological mechanisms linking exposure to PM with diabetes.^4^ In a mouse model, exposure to high concentrations of PM_2·5_ induced insulin resistance and systemic inflammation and increased visceral adiposity in obese study animals.^10^ Ongoing exposure led to impaired glucose tolerance, lower circulating concentrations of adipokines (adiponectin and leptin), and mitochondrial alteration in the same mice.^11^ This and other evidence generated from experimental studies has led to epidemiological studies assessing the relationship between diabetes risk and air pollution.^12^, ^13^, ^14^, ^15^ We aimed to assess the burden of type 2 diabetes attributable to ambient and household PM_2·5_ air pollution using estimates derived from epidemiological studies. We also aimed to evaluate the relative roles of changes in exposure, population size, age, and diabetes incidence in the observed trend in PM_2·5_-attributable diabetes burden. This manuscript was produced as part of the Global Burden of Diseases, Injuries, and Risk Factors Study (GBD) Collaborator Network and in accordance with the GBD Protocol.

## Methods

### Overview

GBD is the most comprehensive global epidemiological study. It estimates the burden of disease from 286 causes of death, 369 diseases and injuries, and 87 risk factors in 204 countries and territories. Risk factors include metabolic, behavioural and environmental factors, such as air pollution.

### Literature review and study extraction

To compile all relevant studies, we followed a two-stage search strategy. In stage 1, we searched PubMed on June 1, 2017, and in 2019 for the most recent meta-analysis or systematic review of studies investigating the effect of ambient air pollution, household air pollution, or second-hand smoke on diabetes. We defined a search string consisting of “diabetes”, “meta-analysis”, or “review” and the exposure indicator (eg, “particulate matter”, “household air pollution”, “indoor air pollution”, or “cooking fuel”). The exact search strings are provided in the appendix p 2). In stage 2, we included additional studies that were identified through other sources, such as those referenced in another study or published and unpublished work sent to us by members of the GBD Collaborator Network.

The primary outcome of interest was type 2 diabetes in adults aged 18 years or older. As some studies did not differentiate between type 1 and type 2, we assumed that cases were dominated by type 2.^2^, ^4^ We included studies that assessed morbidity (ie, incidence) and mortality. We limited our results to case-control and cohort studies and articles written in English. Studies were excluded if the full text could not be obtained, if exposure to PM_2·5_ was short term (eg, several days), or if exposure to active tobacco smoking was not measured in terms of cigarettes per day. Studies that assessed type 1 or gestational diabetes were also excluded. Information from each of the individual studies that were identified, including effect estimates and additional study-specific information, was extracted and used in the meta-regression.

### Exposure assessment

For estimation of ambient air pollution exposure, we used the Data Integration Model for Air Quality (DIMAQ2). This model integrates data from satellite-based measurements of aerosol optical depth, ground measurements from 9960 PM monitoring stations across 108 countries, and chemical transport model simulations.^16^ Global values of PM_2·5_, provided at a 0·1° × 0·1° resolution, were population-weighted to generate mean exposure for each location. Methods and data sources are described in more detail in the appendix (pp 2–5) and have been published elsewhere.^16^

Exposure modelling for household air pollution from solid fuels comprised two components. The first component, the so-called proportional model, estimated the proportion of households using solid cooking fuels, relying on data extracted from multiple national-level and other surveys and the WHO Energy Database.^17^ The second component, the mapping model, estimated the exposure concentration of PM_2·5_ corresponding to solid-fuel use, measured in μg per cubic metre of air (μg/m^3^) for a given location and year based on the Socio-demographic Index. Further information is provided in the appendix (pp 3–4).

### Exposure–response curve

Several studies have derived relative risks over various exposure ranges. These risks differ in magnitude, and the exposure range over which they are assessed varies. Furthermore, at the upper end of the global ambient air pollution exposure range, reliable estimates are not available, due to a scarcity of studies in highly polluted areas. Another challenge in meta-regressing risk estimations from different studies lies in differing study designs and varying adjustment for potential confounders. To account for between-study heterogeneity and to develop a relative risk function that covered the entire global exposure range, we combined risk estimates from all available studies using the newly developed regression tool, MR-BRT (meta-regression—Bayesian, regularised, trimmed).^18^

Risk estimates from studies assessing the impact of ambient air pollution, household air pollution, and second-hand smoke on type 2 diabetes were used to fit a spline, with each risk estimate informing the curve along the study-specific exposure range. Various model settings and priors were tested for fitting the MR-BRT splines. The final models used third-order splines with two interior knots and a constraint on the right-most segment, forcing the fit to be linear rather than cubic at the upper end. We used an ensemble approach to knot placement, wherein 100 different models were run with randomly placed knots and then combined by weighting based on a measure of fit that penalises excessive changes in the third derivative of the curve. Knots were free to be placed anywhere within the 5th and 95th percentile of the data, as long as a minimum width of 10% of that domain existed between them. We included shape constraints so that the risk curves were concave down and monotonically increasing—the most biologically plausible shape for the PM_2·5_ risk curve. On the non-linear segments, we included a Gaussian prior on the third derivative of mean 0 and variance 0·01 to prevent overfitting; on the linear segment, a stronger prior of mean 0 and variance 1 × 10^−6^ was used to ensure that the risk curves did not continue to increase beyond the range of the data.

In addition, we extracted a set of study-specific covariates to explain between-study heterogeneity in risk estimates. We considered whether the study assessed the overall population or a subpopulation using a binary variable; two binary variables indicated whether exposure was measured at the population or individual level and whether exposure was measured at the beginning of the study versus at multiple timepoints during the study period. We also included binary variables indicating whether the exposure or the outcome was self-reported and a variable indicating whether the assessment was blind to exposure. We also accounted for the degree to which study participants were lost to follow-up. Finally, we accounted for control of confounding within each study by creating two covariates. First, we created a binary variable indicating whether the study was randomised. As no studies were randomised, this variable was not included in the meta-regression. In addition, we created a categorical variable indicating the degree of statistical control for potential confounding. This covariate had three levels: the first level indicated that the study had controlled for major confounders—specifically, age, sex, education, income, and smoking status—measured at either the individual or the community level. The next level indicated that the study had controlled for age, sex, and smoking only. The third level indicated that the study controlled only for age and sex but no other confounders. The appendix (pp 9–11) gives a detailed overview over all covariates included in the meta-regression. As none of these covariates was significant in the model, the final model was fitted without them.

### PAF calculation and burden estimation

The population attributable fraction (PAF) quantifies the proportion of cases that can be attributed to the risk factor. That is, PAFs represent the fraction of cases that would be avoided if exposure was reduced to the theoretical minimum risk exposure level. As individuals who are exposed to household sources of PM_2·5_ are also exposed to ambient sources, we used a proportional approach to calculate PAFs for each risk factor. Using the mean annual ambient PM_2·5_ exposure, the proportion of individuals exposed to PM from household solid fuel use, and the value derived from the household PM_2·5_ mapping function for each location and year, we calculated a corresponding relative risk from the exposure–response curve for each 0·1° × 0·1° grid cell. This relative risk was converted to a PAF using the following formula: PAF=(RR–1)/RR, where RR is the relative risk. This PAF, which was initially determined for both sources of PM_2·5_, and was then proportionally attributed to ambient and household. We then aggregated up to the location level (country or administrative unit), weighting by grid cell-level population. By using this strategy, the total PAF is the sum of the ambient and household PAFs, and we assume that both exposures are evenly distributed across different slopes of the exposure–response curve. More details about these calculations can be found in the appendix (p 4). Eventually, the type 2 diabetes burden was determined by multiplying age-specific, sex-specific, year-specific, and location-specific PAFs with deaths, YLLs, YLDs, and DALYs for 204 countries and territories from 1990 to 2019. Over this period, we decomposed the trend in the attributable burden to evaluate the relative roles of changes in exposure, population size, age, and type 2 diabetes incidence. To account for uncertainties in our modelling, we produced 1000 draws of all estimates and intermediate steps. We present estimates (with 95% uncertainty intervals [UIs]) of PAFs and rates per 100 000 population of type 2 diabetes deaths, DALYs, YLDs, and YLLs attributable to PM_2·5_ pollution. We also calculated the PM_2·5_ pollution-deleted DALY rate, which is the expected rate if air pollution were reduced to the theoretical minimum risk exposure level and captures changes in other risk factors or treatment practices.

### Role of the funding source

The funder of the study had no role in study design, data collection, data analysis, data interpretation, or writing of the report.

## Results

**Figure 1:**
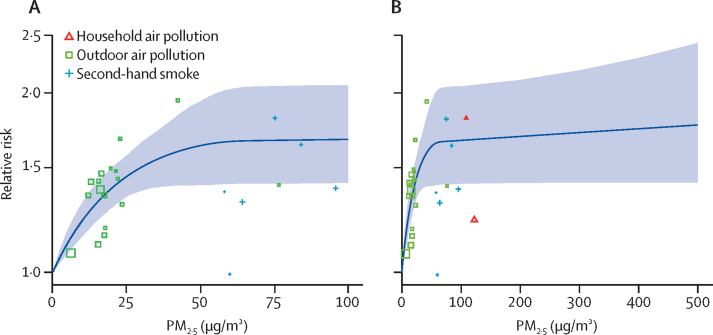
Exposure–response function for PM_2·5_ exposure and type 2 diabetes for an exposure range of 0–100 μg/m^3^ (A) and 0-500 μg/m^3^ (B) The solid line shows the central estimate of the exposure–response curve, and the shaded area depicts 95% uncertainty intervals. The relative risk equals 1 for PM_2·5_ concentrations of 0–2·4 μg/m^3^, which corresponds to the lower bound of the theoretical minimum risk exposure level uncertainty distribution. Each point represents an epidemiological study included in the model. The size of the point reflects the inverse variance used to weight the model. The relative risk axis is on a log scale. PM=particulate matter.

The literature search resulted in 13 studies with 16 effect estimates for ambient air pollution, two sources and effect estimates for household air pollution, and five sources and seven effect estimates for second-hand smoke. Studies assessing the effects of ambient air pollution, second-hand smoke, and household air pollution covered an exposure range of approximately 5–100 μg/m^3^ (annual average). All ambient air pollution, household air pollution, and second-hand smoke studies revealed an increase in type 2 diabetes risk with increased exposure to PM with varying degrees of precision. The estimated relative risk increases rapidly between a PM_2·5_ exposure concentration of 5 μg/m^3^ and approximately 50 μg/m^3^; above this concentration, the curve flattens and shows only minimal increases (figure 1).

**Figure 2:**
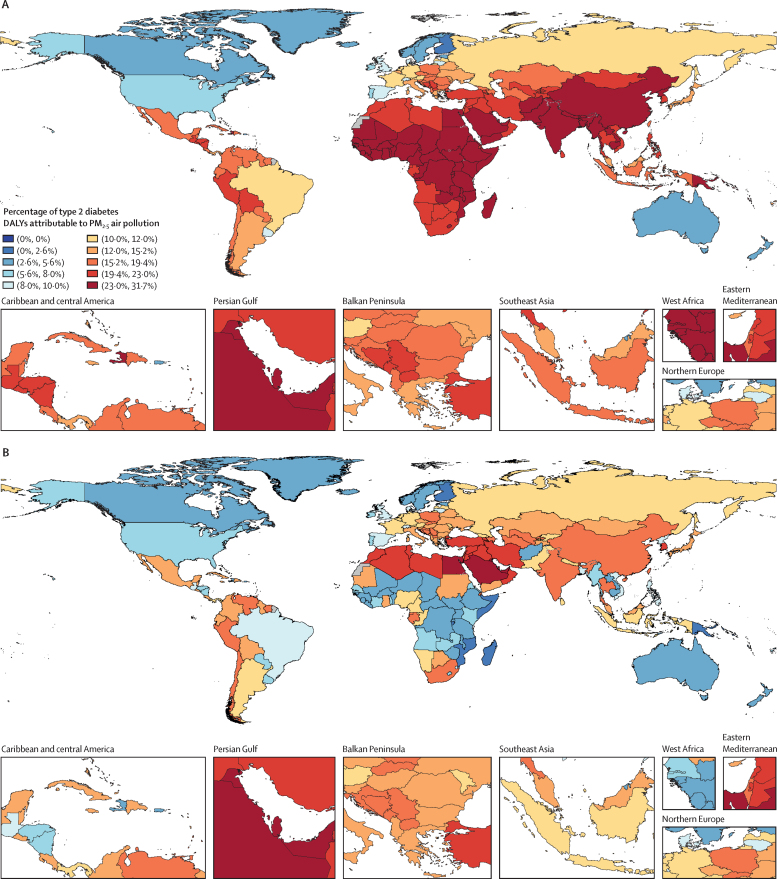

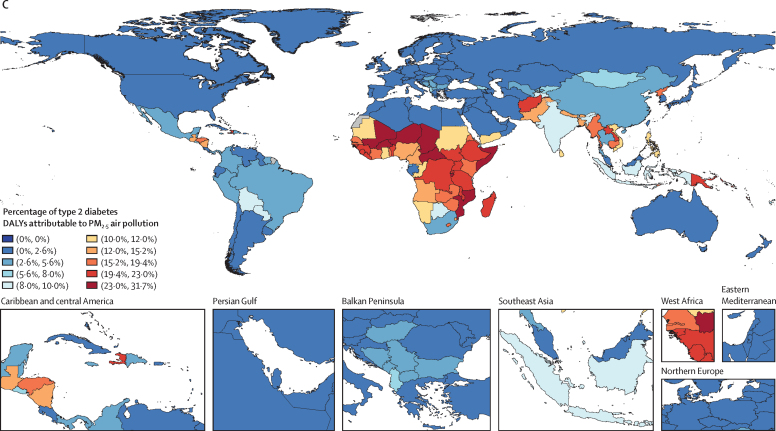
Spatial distribution of the PAF of type 2 diabetes DALYs at all ages and in both sexes attributable to PM_2·5_ air pollution, 2019 Maps show the PAF of type 2 diabetes DALYs at all ages and in both sexes in 2019 that was due to PM_2·5_ air pollution (A), ambient PM_2·5_ pollution (B), and household air pollution from solid fuels (C). DALYs=disability-adjusted life-years. PAF=population attributable fraction. PM=particulate matter.

In 2019, about a fifth of the total type 2 diabetes burden was attributable to air pollution, with a death rate of 3·78 (95% UI 2·68–4·83) per 100 000 population and a DALY rate of 167 (117–223) per 100 000 population (table). 13·4% (9·49–17·5) of type 2 diabetes deaths were due to ambient air pollution, whereas 6·50% (4·22–9·53) were due to household air pollution. Africa, the Middle East, and south and east Asia exhibited a particularly high PM_2·5_-attributable burden (figure 2A). Noticeably, ambient air pollution contributed to a large extent of this burden in north Africa and the Middle East, whereas in sub-Saharan Africa, most of the air pollution-attributable burden stemmed from household air pollution (figures 2B, 2C). North America, Australia, and Scandinavia showed distinctly low air-pollution-attributable type 2 diabetes burden (figure 2). YLDs showed a greater increase than YLLs; this trend could be observed globally, but especially in north Africa and the Middle East and in south Asia (table).

**Table:** Type 2 diabetes deaths, DALYs, YLDs, and YLLs attributable to all PM_2·5_ air pollution, ambient PM_2·5_ pollution, and household PM_2·5_ pollution from solid fuels in seven GBD super-regions and globally in 2019, and change from 1990 to 2019

	**Deaths**			**DALYs**			**YLDs**			**YLSs**		
	**Percentage (%)**	**Rate per 100 000**	**Percentage change (%)**	**Percentage (%)**	**Rate per 100 000**	**Percentage change (%)**	**Percentage (%)**	**Rate per 100 000**	**Percentage change (%)**	**Percentage (%)**	**Rate per 100 000**	**Percentage change (%)**
**PM_2·5_ air pollution**												
Global	19·9% (14·2 to 25·5)	3·78 (2·68 to 4·83)	54·8% (25·0 to 75·1)	19·6% (13·9 to 25·0)	167 (117 to 223)	64·2% (32·7 to 82·5)	18·9% (13·5 to 24·2)	86·0 (52·1 to 128)	90·0% (56·9 to 108)	20·2% (14·5 to 25·9)	81·5 (58·4 to 104)	43·6% (15·8 to 63·2)
Central Europe, eastern Europe, and central Asia	16·2% (11·2 to 21·6)	2·52 (1·72 to 3·36)	67·5% (37·8 to 100)	15·9% (10·9 to 21·2)	135 (85·7 to 190)	59·7% (33·5 to 91·7)	15·6% (10·7 to 20·8)	83·2 (47·4 to 125)	64·6% (37·4 to 99·5)	16·4% (11·4 to 21·8)	51·6 (35·5 to 68·8)	52·4% (26·1 to 82·6)
High income	9·62% (5·76 to 14·4)	1·91 (1·14 to 2·84)	−19·0% (−41·0 to 15·2)	9·30% (5·47 to 14·2)	93·2 (52·1 to 149)	7·31% (−24·4 to 47·9)	9·26% (5·44 to 14·1)	63·9 (32·4 to 106)	43·2 (2·15 to 101)	9·38% (5·56 to 14·2)	29·3 (17·6 to 44·3)	−30·6% (−49·9 to −2·48)
Latin America and Caribbean	16·0% (11·2 to 21·0)	5·46 (3·75 to 7·21)	49·8% (22·0 to 80·3)	16·0% (11·3 to 21·0)	218 (147 to 297)	52·9% (25·3 to 83·6)	15·9% (11·1 to 20·8)	101 (59·6 to 154)	81·5% (50·8 to 117)	16·1% (11·4 to 21·2)	117 (80·5 to 154)	34·6% (9·38 to 62·7)
North Africa and Middle East	22·4% (16·2 to 27·9)	3·51 (2·53 to 4·54)	47·4% (27·3 to 70·4)	22·3% (16·2 to 27·8)	176 (124 to 237)	89·2% (64·8 to 108)	22·2% (16·1 to 27·6)	97·3 (58·6 to 146)	161% (128 to 175)	22·5% (16·3 to 28·0)	79·0 (57·2 to 103)	41·2% (20·1 to 65·0)
South Asia	24·5% (17·6 to 31·4)	4·59 (3·24 to 5·98)	98·0% (39·5 to 149)	24·3% (17·5 to 31·2)	203 (140 to 275)	98·2% (42·0 to 134)	24·3% (17·4 to 31·2)	105 (64·2 to 155)	122% (59·0 to 149)	24·4% (17·6 to 31·2)	98·8 (70·3 to 129)	78·0% (24·8 to 127)
Southeast Asia, east Asia, and Oceania	21·6% (15·7 to 27·0)	4·05 (2·91 to 5·14)	89·5% (47·5 to 122)	21·8% (15·9 to 27·3)	187 (134 to 244)	87·8% (47·1 to 112)	22·3% (16·2 to 28·0)	97·3 (59·3 to 143)	110% (65·4 to 133)	21·3% (15·5 to 26·7)	89·9 (64·8 to 114)	68·4% (30·7 to 98·9)
Sub-Saharan Africa	24·4% (17·3 to 33·7)	3·51 (2·42 to 4·80)	−8·38% (−33·3 to 13·0)	24·2% (17·1 to 33·6)	122 (83·5 to 173)	−2·36% (−28·7 to 18·8)	24·1% (17·1 to 33·6)	40·8 (24·1 to 62·8)	30·5% (−2·38 to 48·3)	24·2% (17·1 to 33·5)	81·6 (56·1 to 112)	−13·3% (−37·2 to 9·10)
**Ambient PM**_2·5_**pollution**												
Global	13·4% (9·49 to 17·5)	2·54 (1·76 to 3·34)	144% (104 to 196)	13·6% (9·73 to 17·9)	117 (79·3 to 158)	168% (126 to 225)	13·9% (9·94 to 18·1)	63·3 (37·6 to 95·9)	203% (157 to 269)	13·3% (9·47 to 17·4)	53·5 (37·3 to 70·1)	136% (96·9 to 186)
Central Europe, eastern Europe, and central Asia	14·4% (9·83 to 19·2)	2·23 (1·52 to 3·01)	105% (58·5 to 180)	14·2% (9·73 to 19·0)	120 (75·9 to 172)	93·0% (52·2 to 161)	14·0% (9·54 to 18·8)	74·7 (42·2 to 114)	96·6% (55·5 to 167)	14·5% (10·0 to 19·4)	45·7 (31·4 to 61·3)	87·4% (45·1 to 155)
High income	9·53% (5·71 to 14·3)	1·89 (1·13 to 2·82)	−16·7% (−40·1 to 20·8)	9·23% (5·44 to 14·0)	92·5 (51·9 to 148)	10·1% (−23·2 to 54·6)	9·20% (5·40 to 14·0)	63·4 (32·0 to 104)	46·4% (3·82 to 108)	9·29% (5·52 to 14·0)	29·0 (17·3 to 43·8)	−28·6% (−48·7 to 1·59)
Latin America and Caribbean	12·1% (8·27 to 16·2)	4·12 (2·73 to 5·61)	124% (66·5 to 217)	12·2% (8·33 to 16·3)	166 (108 to 233)	129% (71·4 to 222)	12·2% (8·38 to 16·4)	78·0 (44·5 to 120)	177% (104 to 295)	12·1% (8·29 to 16·2)	87·7 (58·9 to 119)	98·8% (47·4 to 182)
North Africa and Middle East	20·9% (15·2 to 26·2)	3·28 (2·36 to 4·22)	104% (71·7 to 151)	20·7% (15·0 to 25·9)	164 (117 to 220)	164% (127 to 215)	20·7% (15·0 to 26·0)	90·6 (54·3 to 136)	272% (229 to 339)	20·8% (15·2 to 26·0)	73·1 (52·9 to 94·9)	94·2% (62·8 to 141)
South Asia	15·2% (10·5 to 20·5)	2·85 (1·90 to 3·88)	484% (250 to 1130)	15·2% (10·5 to 20·5)	128 (84·4 to 177)	471% (251 to 1070)	15·3% (10·5 to 20·5)	66·0 (38·1 to 102)	526% (292 to 1190)	15·2% (10·4 to 20·5)	61·6 (41·1 to 83·5)	422% (212 to 1000)
Southeast Asia, east Asia, and Oceania	14·3% (10·0 to 18·9)	2·69 (1·85 to 3·58)	357% (203 to 660)	15·2% (10·7 to 19·9)	130 (85·6 to 178)	359% (204 to 675)	16·5% (11·8 to 21·4)	71·9 (42·4 to 108)	423% (241 to 817)	13·8% (9·59 to 18·3)	58·3 (40·1 to 78·2)	298% (164 to 561)
Sub-Saharan Africa	8·70% (5·62 to 12·5)	1·25 (0·792 to 1·80)	139% (74·1 to 231)	8·24% (5·25 to 11·9)	41·6 (26·5 to 61·2)	149% (84·0 to 243)	7·93% (4·98 to 11·5)	13·4 (7·43 to 21·0)	211% (132 to 324)	8·39% (5·36 to 12·1)	28·3 (17·8 to 41·6)	127% (65·7 to 216)
**Household air pollution from solid fuels**												
Global	6·50% (4·22 to 9·53)	1·24 (0·782 to 1·79)	−11·5% (−35·6 to 12·5)	5·92% (3·81 to 8·64)	50·7 (31·4 to 75·6)	−13·3% (−35·9 to 10·5)	5·00% (3·16 to 7·44)	22·7 (12·2 to 37·6)	−6·79% (−30·5 to 17·8)	6·95% (4·51 to 10·1)	28·0 (17·6 to 40·7)	−17·9% (−40·0 to 4·07)
Central Europe, eastern Europe, and central Asia	1·84% (0·794 to 3·55)	0·286 (0·121 to 0·544)	−31·2% (−56·8 to −2·18)	1·71% (0·721 to 3·36)	14·5 (5·83 to 29·1)	−34·5% (−58·3 to −7·33)	1·60% (0·66 to 3·26)	8·53 (3·20 to 18·7)	−32·2% (−57·9 to −3·14)	1·88% (0·815 to 3·61)	5·93 (2·55 to 11·2)	−37·5% (−59·4 to −12·2)
High income	0·086% (0·028 to 0·212)	0·017 (0·006 to 0·041)	−79·8% (−88·0 to −70·0)	0·072% (0·023 to 0·180)	0·724 (0·224 to 1·77)	−74·7% (−85·0 to–62·5)	0·065% (0·020 to 0·161)	0·449 (0·129 to 1·12)	−65·2% (−79·1 to −48·2)	0·088% (0·029 to 0·217)	0·275 (0·091 to 0·668)	−82·6% (−89·7 to −73·9)
Latin America and Caribbean	3·93% (2·38 to 6·01)	1·34 (0·789 to 2·05)	−25·6% (−46·6 to −3·44)	3·84% (2·34 to 5·91)	52·3 (30·6 to 80·7)	−25·6% (−45·8 to −4·01)	3·62% (2·17 to 5·62)	23·1 (11·7 to 38·5)	−16·1% (−38·5 to 7·98)	4·04% (2·47 to 6·16)	29·2 (17·5 to 44·2)	−82·6% (−89·7 to −73·9)
North Africa and Middle East	1·48% (0·860 to 2·35)	0·233 (0·135 to 0·382)	−69·9% (−78·7 to −59·1)	1·59% (0·971 to 2·42)	12·6 (7·42 to 19·7)	−59·7% (−67·7 to −49·3)	1·52% (0·939 to 2·22)	6·64 (3·48 to 11·1)	−48·3% (−56·8 to −38·8)	1·68% (0·969 to 2·75)	5·93 (3·35 to 10·0)	−67·6% (−77·7 to −55·4)
South Asia	9·27% (5·75 to 13·8)	1·74 (1·07 to 2·65)	−5·06% (−38·1 to 32·9)	9·07% (5·60 to 13·6)	75·9 (44·9 to 118)	−5·54% (−36·9 to 30·2)	8·95% (5·56 to 13·4)	38·7 (20·2 to 63·3)	5·58% (−28·5 to 43·5)	9·19% (5·68 to 13·7)	37·2 (22·9 to 56·2)	−14·8% (−44·8 to 20·1)
Southeast Asia, east Asia, and Oceania	7·25% (4·27 to 11·0)	1·36 (0·803 to 2·07)	−12·1% (−40·7 to 22·9)	6·64% (3·86 to 10·2)	57·0 (32·3 to 89·5)	−20·1% (−47·1 to 10·9)	5·82% (3·31 to 9·24)	25·4 (12·5 to 43·8)	−22·1% (−50·6 to 11·1)	7·48% (4·45 to 11·3)	31·6 (18·6 to 48·1)	−18·4% (−44·8 to 13·2)
Sub-Saharan Africa	15·7% (10·7 to 23·9)	2·26 (1·48 to 3·41)	−31·6% (−49·8 to −13·5)	16·0% (10·8 to 24·3)	80·8 (52·0 to 125)	−25·6% (−45·6 to −6·84)	16·2% (11·0 to 24·6)	27·4 (15·3 to 45·0)	1·65% (−23·1 to 20·3)	15·8% (10·7 to 24·1)	53·4 (34·6 to 80·6)	−34·6% (−52·8 to −15·6)

Data in parentheses are 95% uncertainty intervals. Data are presented to three significant figures. DALYs=disability-adjusted life-years. GBD=Global Burden of Diseases, Injuries, and Risk Factors Study. PM=particulate matter. YLDs=years lived with disability. YLLs=years of life lost.

**Figure 3:**
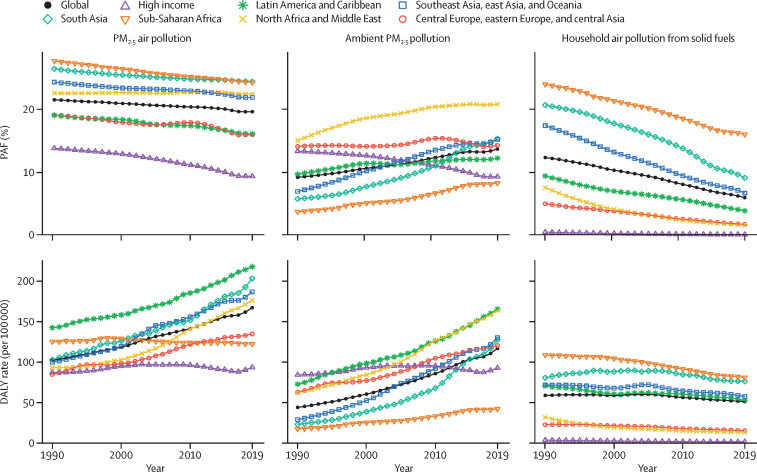
Temporal trend in the type 2 diabetes burden attributable to air pollution, ambient particulate matter pollution, and household air pollution from solid fuels, 1990–2019 Data are for all ages and both sexes by GBD super-region and globally. DALYs=disability-adjusted life-years. GBD=Global Burden of Diseases, Injuries, and Risk Factors Study. PAF=population attributable fraction. PM=particulate matter.

Globally, we observed marked increases in the rate of type 2 diabetes from 1990 to 2019.^3^ This pattern was observed for most regions, with the exception of high-income regions, central Europe, eastern Europe, and central Asia, for which rates were stagnant after 2010 (figure 3). Rates of type 2 diabetes attributable to ambient air pollution showed a similar pattern as for overall air pollution, whereas rates of type 2 diabetes attributable to household air pollution either showed a slight decline or stagnation. With regard to the time trend in the PAF of type 2 diabetes attributable to air pollution, we found a decrease in all super-regions from 1990 to 2019. This decline was driven by declines in PAFs in household air pollution. PAFs for ambient air pollution increased in most regions, except for high-income regions, where we observed a decrease. In central Europe, eastern Europe, and central Asia, PAFs for ambient air pollution were mostly stagnant (figure 3).

**Figure 4:**
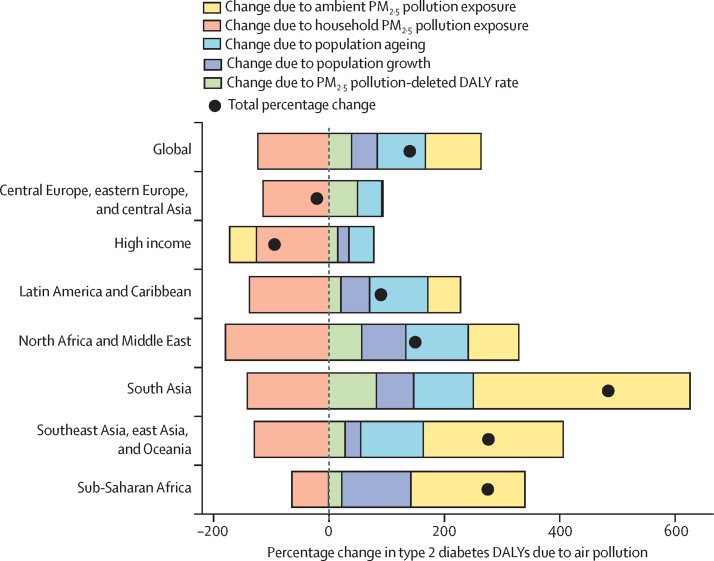
Drivers of trends in type 2 diabetes DALYs attributable to air pollution in GBD super-regions and globally, 1990–2019 Data are for all ages and both sexes. The PM_2·5_ pollution-deleted DALY rate is the expected rate if air pollution were reduced to the theoretical minimum risk exposure level and captures changes in other risk factors or treatment practices. DALYs=disability-adjusted life-years. GBD=Global Burden of Diseases, Injuries, and Risk Factors Study. PM=particulate matter.

Figure 4 shows a decomposition of individual factors driving changes in type 2 diabetes DALYs attributable to air pollution between 1990 and 2019. A reduction in exposure to household air pollution led to a decrease in the attributable burden in all considered regions as well as globally. In high-income countries, improvements in ambient air pollution levels contributed to reduced attributable risks, whereas in all other regions we observed an increase in DALYs due to ambient PM_2·5_ exposure. In Latin America and the Caribbean, southeast Asia, east Asia, and Oceania, and south Asia, where improvement in household air pollution led to the biggest reductions in the burden, these reductions were vastly offset by increases in ambient air pollution exposure. A similar pattern, but with slightly smaller numbers, was observed in north Africa and the Middle East and sub-Saharan Africa. In all GBD super-regions, PM_2·5_-deleted DALY rates—ie, changes in other risk factors—drove increases in type 2 diabetes. Population ageing and growth substantially contributed to increases in the type 2 diabetes burden attributable to PM_2·5_. Although population ageing played a major part, in high-income countries, central and eastern Europe, and central Asia, in sub-Saharan Africa, population growth drove most of the air pollution-attributable burden. We observed a net increase in air pollution-attributable type 2 diabetes DALYs in all super-regions, with a global increase of approximately 140%.

## Discussion

This study provides a systematic analysis of the type 2 diabetes burden attributable to ambient PM_2·5_ pollution, composed of ambient air pollution and household air pollution. Ambient PM_2·5_ ranked as the third leading risk factor for type 2 diabetes within the GBD hierarchy, after high fasting plasma glucose and high body-mass index.^17^ Approximately a fifth of the global burden of type 2 diabetes was attributable to air pollution, with 13·4% from ambient PM_2·5_ and 6·5% from household air pollution. At the population level, air pollution was responsible for more attributable burden than either tobacco or physical inactivity.^17^ Combining the exposure–response relationship with global location-specific exposure data revealed a geographically explicit pattern. Africa, Asia, and South America in particular showed a high burden attributable to air pollution. In most areas, with the exception of sub-Saharan Africa, this burden was dominated by ambient PM_2·5_, whereas household air pollution played only a minor part. Over the past three decades, absolute DALYs and DALY rates attributable to PM_2·5_ pollution have considerably increased. Our decomposition analysis showed that in most regions, improvements in household air pollution were counterbalanced by increased ambient air pollution. In addition to increased ambient air pollution exposure, population growth and ageing contributed to the large increase in the type 2 diabetes burden attributable to air pollution.

Our study relied on an extensive number of studies and effect estimates that were combined and integrated across the exposure ranges. Based on the exposure–response curve, we estimated the attributable burden for ambient air pollution and household air pollution from 1990 to 2019 and did a decomposition analysis. Despite these obvious strengths, there are several limitations, and causality and confounding especially need to be addressed. Although cohort and case-control studies, the two types of epidemiological studies compiled for this research, rank at the upper end of the epidemiological evidence hierarchy, they are by no means proof of causality. Studies assessing the relationship between air pollution and type 2 diabetes consistently revealed increased incidence and prevalence. Most of the studies adjusted for personal-level confounders such as age and sex. Some, but not all, studies adjusted for socioeconomic status (eg, income, education and body-mass index) and behaviour (eg, smoking, alcohol use, and physical activity). Several studies have suggested that the effect of air pollution on type 2 diabetes varied over different groups. Honda and colleagues^19^ found increased risk in a cohort of older people (age ≥57 years), and other studies particularly highlighted women as more vulnerable.^20^ Park and colleagues^20^ emphasised the likeliness of different population groups and ethnicities exhibiting different risks. Similarly, other forms of ambient air pollution not considered in our and other studies, such as gaseous pollutants, might also be relevant. Some evidence suggests that traffic-related air pollution is a higher risk for type 2 diabetes.^21^, ^22^, ^23^ Several studies have found a consistent effect of nitrogen dioxide on type 2 diabetes incidence, but this effect is less consistent for PM.^7^, ^24^ However, few studies adjust for nitrogen dioxide or noise, which are often correlated.^25^

The exposure–response relationship sharply increases from the theoretical minimum risk exposure level to approximately 50 μg/m^3^ and levels off throughout the exposure range. Although the shape fits the data well, the constraints and priors imposed on the fit enable this shape. Specifically, we included shape priors so that the curve would be monotonically increasing and concave. Furthermore, a strong prior on the upper segment prevented the curve from strongly increasing beyond the exposure range for which effect estimates were available. The shape of our derived exposure–response curve, which reflects a strong increase at lower exposures and a levelling off at higher air pollution levels, has been found in other studies, such as the hazard curve developed from the US Veterans Cohort data.^26^ This suggests the possibility of saturation of the mechanism (or mechanisms) driving the biological connection between PM exposure and type 2 diabetes, as has been postulated for cardiovascular mortality.^27^, ^28^ Indeed, the same non-linearity has been reported for cardiovascular mortality in the Canadian Census and cohort studies in males in China.^24^, ^29^ In addition to saturation, changes in the aerosol composition might be driving this shape. The issue of equitoxicity has frequently been raised within the scientific community. Although differing toxicity is plausible, so far, PM_2·5_ has proved to be the most consistent and robust predictor of mortality in studies of long-term exposure.^30^, ^31^, ^32^

In summary, the relationship between air pollution and type 2 diabetes is highly complex, and effect estimations are strongly affected by study design, cohort characteristics, the degree of covariate adjustment, and exposure assessment. Although our meta-regression framework cannot fully overcome all limitations in the original study designs, the MR-BRT tool allowed us to account for several study-level covariates and remaining between-study heterogeneity. The exposure–response relationship was significant along the entire range of exposures. In conjunction with the biological plausibility of an effect of PM inhalation on type 2 diabetes, we considered the evidence sufficient to include PM_2·5_ and type 2 diabetes as a new risk–outcome pair into the GBD. Our results highlight the relevance of air pollution as a risk factor for type 2 diabetes. The attributable burden shows strong regional variation and distinct temporal trends: although ambient air pollution contributes a larger share globally, in sub-Saharan Africa, a larger part can be attributed to household air pollution. In almost all GBD super-regions, except the high-income region, the burden attributable to ambient air pollution has increased, whereas the burden attributable to household air pollution has decreased globally and in all regions.

## Data sharing

To download the data used in these analyses, please visit the Global Health Data Exchange GBD 2019 results website

## Declaration of interests

T W Bärnighausen reports support for the present manuscript from the Alexander von Humboldt Foundation, Wellcome Trust, German Research Foundation, and US National Institutes of Health (National Institute of Allergy and Infectious Diseases, National Institute on Aging, and Fogarty International Center), all paid to his institution. Y Béjot reports consulting fees from Novo Nordisk and honoraria for lectures from Medtronic, Boehringer Ingelheim, Pfizer, Bristol Myers Squibb (BMS), Servier Laboratories, and Amgen, all outside the submitted work. L A Càmera reports non-financial support for the present manuscript from Sociedad Argentina de Medicina and Hospital Italiano Buenos Aires. X Dai reports support for the present manuscript via salary from the Institute for Health Metrics and Evaluation University of Washington. I Filip and A Radfar report support from Avicenna Medical and Clinical Research Institute, outside the submitted work. J J Jozwiak reports payment via personal fees for lectures, presentations, speaker's bureaus, manuscript writing or educational events from Teva Pharmaceuticals, Amgen, Synexus, Boehringer Ingelheim, Zentiva, and Sanofi, all outside the submitted work. M Kivimäki reports research grants to University College London (London, UK) from the Wellcome Trust (221854/Z/20/Z) and the UK Medical Research Council (MR/S011676/1), outside the submitted work. S Lorkowski reports (all outside the submitted work) grants or contracts paid to his institution from Akcea Therapeutics Germany; consulting fees from Danone, Novartis Pharma, Swedish Orphan Biovitrum (SOBI), and Upfield; payment or honoraria for lectures, presentations, speaker's bureaus, manuscript writing, or educational events from Akcea Therapeutics Germany, Amarin Germany, Amedes Holding, Amgen, Berlin-Chemie, Boehringer Ingelheim, Daiichi Sankyo Deutschland, Danone, Hubert Burda Media Holding, Janssen-Cilag, Lilly Deutschland, Novartis, Novo Nordisk, F Hoffmann-La Roche (Roche), Sanofi-Aventis, SYNLAB Holding Deutschland, and SYNLAB Akademie; support for attending meetings or travel from Amgen; and participation on a data safety monitoring board or advisory board for Akcea Therapeutics Germany, Amgen, Daiichi Sankyo Deutschland, Novartis, and Sanofi-Aventis. P W Mahasha reports (all outside the submitted work) participation on a data safety monitoring board or advisory board as a member of the Technical Advisory Panel to the Office of Health Standards Compliance and Health Ombud from Feb 12, 2020, to Feb 11, 2022, and leadership or fiduciary roles in boards, societies, committees, or advocacy groups, paid or unpaid, as a member of the Federation of Infectious Diseases Societies of Southern Africa since November, 2021; the EU-Africa PerMed Consortium from February, 2021, to January, 2025; the South African Society for Biochemistry and Molecular Biology since August, 2021; the International Society for Infectious Diseases since November, 2021; the South African Society of Microbiology since November, 2021; the COVID-19 Clinical Research Coalition since August, 2021; the Scholars Academic and Scientific Society since August, 2021; Cochrane International since October, 2019 (renewed on March 13, 2022); and the South African Council for Natural Scientific Professions since June, 2018. A Ortiz reports (all outside the submitted work) grants from Sanofi; consultancy or speaker fees or travel support from Advicenne, Astellas, AstraZeneca, Amicus, Amgen, Fresenius Medical Care, GlaxoSmithKline, Bayer, Sanofi-Genzyme, Menarini, Kyowa Kirin, Alexion, Idorsia, Chiesi, Otsuka, Novo-Nordisk, and Vifor Fresenius Medical Care Renal Pharma; is Director of the Catedra Mundipharma-UAM of diabetic kidney disease and the Catedra AstraZeneca-UAM of chronic kidney disease and electrolytes; is the Editor-in-Chief for *Clinical Kidney Journal*; and reports a leadership or fiduciary role with the European Renal Association and Madrid Renal Association. P A Mahesh reports grants or contracts paid to his institution from the Government of India (Wellcome Trust/DBT team science grant), the US National Institutes of Health Global Infectious Diseases, and the Swedish Heart Lung Foundation, outside the submitted work. M J Postma reports grants paid to his institution from Boehringer Ingelheim and AstraZeneca, and stock or stock options in Health-Ecore (25%) and Pharmacoeconomics Advice Groningen (100%), all outside the submitted work. J Sanabria reports a pending patent for pNaKtide, and leadership or fiduciary roles in boards, societies, committees, or advocacy groups, paid or unpaid with the Society for the Surgery of the Alimentary Tract, American Association for the Study of Liver Diseases, Society of University Surgeons, and American Society of Transplant Surgeons, all outside the submitted work. P R Valdez reports support for the present manuscript from Sociedad Argentina de Medicina and Hospital Velez Sarsfield Buenos Aires. All other authors declare no competing interests.
