# Supplementary material for: Estimates, trends, and drivers of the global burden of type 2 diabetes attributable to PM2·5 air pollution, 1990–2019: an analysis of data from the Global Burden of Disease Study 2019
Source: Lancet Planet Health. 2022 Jul 6;6(7):e586–600. doi: 10.1016/S2542-5196(22)00122-X (PMC9278144; doi:10.1016/S2542-5196(22)00122-X)
Supplement: Supplementary appendix [file mmc1.pdf]

THE LANCET
Planetary Health

## Supplementary appendix

This appendix formed part of the original submission and has been peer reviewed. We post it as supplied by the authors.

Supplement to: GBD 2019 Diabetes and Air Pollution Collaborators. Estimates, trends, and drivers of the global burden of type 2 diabetes attributable to $PM_{2.5}$ air pollution, 1990–2019: an analysis of data from the Global Burden of Disease Study 2019. *Lancet Planet Health* 2022; **6:** e586–600.

# Table of Contents


Literature Review and Search Strings....2
Input data and modeling strategy....2
Ambient Air Pollution Exposure....2
Household Air Pollution Exposure....3
Proportion Model....3
PM2.5 Mapping Model....3
Theoretical minimum-risk exposure level....4
Exposure assessment/conversion in smoking studies....4
Proportional PAF calculations....4
References:....6
Tables....7

# Literature Review and Search Strings

To build the joint exposure-response curve we began with a literature review of each of the four sources of particulate matter exposure—ambient air pollution (AAP), household air pollution, (HAP), and secondhand smoke (SHS)—and diabetes. In stage 1, we conducted a PubMed search for the most recent meta-analysis or systematic review for each of the four sources. In stage 2, we found additional studies through searches of the literature or collaborators' knowledge of published and unpublished work. The search strategy and resulting studies are featured in Supplementary Figure S1.

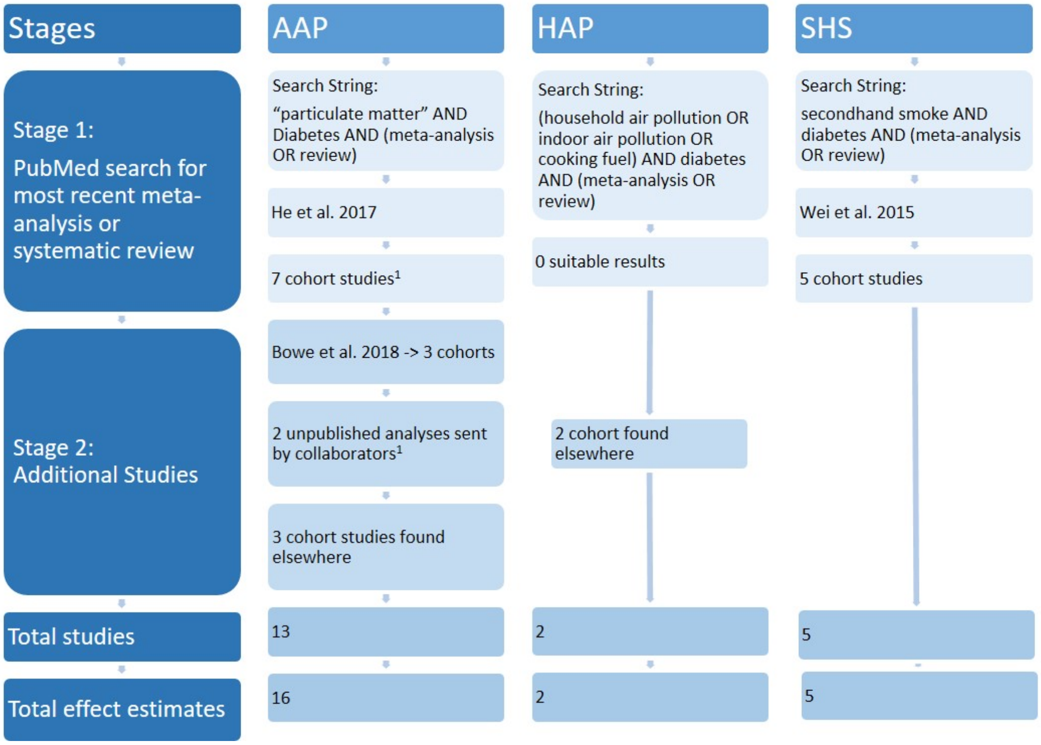


*Supplementary Figure S1: Flow chart outlining different stages of literature search identifying relevant studies for inclusion into meta-analysis and IER*

# Input data and modeling strategy

## Ambient Air Pollution Exposure

Briefly, we estimated exposure to ambient particulate matter pollution as the population-weighted annual mean concentration of particles less than 2.5 micrometers in diameter ($PM_{2.5}$), measured in μg/$m^3$. input data include satellite measurements of aerosol optical depth described in van Donkelaar et al. 2016[1], PM measurements from ground monitoring stations from the WHO Global Ambient Air Quality Database[2], chemical transport models, land-use data, and population estimates.

For more information about ambient air pollution exposure definition, input data sources, and modelling strategy, see Supplementary Appendix 1 of the GBD 2017 Risk Factor Capstone paper[3], Section 4.4, pages 67-71. Additionally, the modelling approach, known as the Data Integration Model for Air Quality (DIMAQ), is detailed in Shaddick et al. 2016[4] and Shaddick et al. 2018[5]

## Household Air Pollution Exposure

Exposure to household air pollution from solid fuels (HAP) has two fundamental components. The first is estimating the proportion of households using solid cooking fuels. We will call this the Proportion Model. The definition of solid fuel in our analysis includes coal, wood, charcoal, dung, and agricultural residues. The second component of the modeling involves estimating the exposure level of PM2.5 corresponding to solid-fuel use and measured in $\mu g/m^3$ for a given location and year. We will call that the PM2.5 Mapping Model.

### Proportion Model

#### *Input data*

Data estimating the percentage of individuals using solid cooking fuels came from international survey series such as Demographic and Health Surveys (DHS) and Living Standards Measurement Surveys (LSMS) and country-specific series such as the South Africa General Household Survey. We also included additional HAP estimates from the WHO Energy Database[6].

#### *Modelling strategy*

Because we do not have data for all locations and years, we used a common GBD modelling tool to fill in the gaps. We modeled this data at the individual level using a three-step modelling strategy that uses linear regression, spatiotemporal regression, and Gaussian Process Regression (ST-GPR). The ST-GPR modelling process is detailed in Supplementary Appendix 1 of the GBD 2017 Risk Factor Capstone paper[3], Section 2.3.3, pages 28-33. The first step is a mixed-effect linear regression of logit-transformed proportion of households using solid cooking fuels. The linear model includes fixed effects on maternal education and the proportion of the population living in urban areas and nested random effects on GBD region and GBD super-region with the following model formula:

$$data \sim maternal\_educ\_yrs\_pc + prop\_urban + (1|region) + (1|super\_region)$$

After ST-GPR we have estimates of the proportion of individuals using solid fuel for every location and year.

### PM2.5 Mapping Model

Not all who use solid fuel for cooking experience the same level of PM2.5 exposure. The PM2.5 mapping model estimates the level of exposure in $\mu g/m^3$ associated with using solid fuel in a given location and year. We use the WHO Global database of household energy measurements[7] and create our model based upon previously published work[8]. The database used for modeling in GBD 2017 contains about 90 studies from 41 unique GBD locations in 19 countries. In these studies researchers measured PM2.5 levels among individuals who used various fuel types for cooking with personal or kitchen monitors. Because these measurements capture PM2.5 exposure from both ambient and household sources, we first subtracted off the GBD estimated ambient exposure level for the given location and year to get the "excess" PM2.5 due to solid fuel use.

We ran a model with the following formula, where *PM2.5* is the excess PM2.5 exposure (study measurement – ambient exposure level), *sdi* is the socio-demographic index for the given study location and year, and *monitor_loc, measure_std,* and *non_solid* are binary indicators of whether the monitoring was in the kitchen or using a personal monitor, whether the measurement took place over at least 48 hours, and whether the household used solid or non-solid fuels respectively:

$$\log(PM2.5) \sim sdi + monitor\_loc + measure\_std + non\_solid$$

We included the *monitor_loc* and the *measure_std* variables to account for systematic biases in study design and assumed a gold standard of personal monitoring and measurements over at least 48 hours. *Non_solid* is included because some studies took measurements of households using solid fuel and households using electricity or gas. We predicted out for all GBD locations and years based on sdi.

Finally, due to traditional gender roles and time spent indoors, studies show that women and children experience higher exposure levels due to household air pollution than men. To account for this in the model, we used seven studies, which reported personal measurements separately for men, women, and children, to generate ratios. These ratios were used to scale the PM2.5 mapping model accordingly. The scaled estimates were used in the proportional PAF calculations to determine the exposure level to obtain the RR from the IER.

## Theoretical minimum-risk exposure level

The TMREL of the IER is estimated as a uniform distribution between 2.4 and 5.9 ug/m$^3$ and is based on observed exposures in several North American cohorts. This is described in more detail in Supplementary Appendix 1 of the GBD 2017 Risk Factor Capstone paper[3], Section 4.4, page 73.

## Exposure assessment/conversion in smoking studies

AS exposure is measured in categories of cigarettes-per-day such as 5-10 cigarettes-per-day. We converted this exposure to a $PM_{2.5}$ value by taking the midpoint of the range and multiplying it by a conversion factor of 667 microgram/m$^3$ per cigarette, according to previous work and based on the following assumptions: average breathing rate of 18 m^3/day and inhaled dose of 12,000 micrograms PM2.5 mass per cigarette[9].

We use two sources to estimate the PM2.5 exposure level for a given SHS study. The first is estimated number of cigarettes smoked per smoker per day for every location and year, 1980 through 2017. Secondly, a study in Sweden measured the PM2.5 exposure in homes of smokers[10]. We divided the household PM2.5 exposure level by the average number of cigarettes smoked per smoker per day in Sweden over the study duration to estimate the SHS PM2.5 exposure per cigarette (2.31 (95% U.I. 1.53, 3.39). For each of the seven SHS and Diabetes studies, we multiplied the estimated number of cigarettes per smoker per day by the average PM2.5 exposures per cigarette to generate a predicted PM2.5 exposure level.

## Proportional PAF calculations

Let $p_0$ be the proportion of the population exposed only to Ambient pollution and $p_1$ be the proportion of the population exposed to both ambient air pollution and household air pollution from solid fuel for cooking such that $\mathrm{p}_0 + p_1 = 1$.

Let $z_0$ be the mean exposure of ambient $PM_{2.5}$, $z_1$ be the additiClosedonal $PM_{2.5}$ exposure due to solid-fuel use, and $z_{cf}$ be the counterfactual level of $PM_{2.5}$ exposure defined by the TMREL. The average population exposure, denoted $z_{mean}$, is a weighted average of exposures calculated as $z_0 + (\mathrm{p}_1 * z_1)$.

The risk function is described elsewhere and is denoted as $IER(z)$ which is equal to the RR at a given exposure level, $z$.

Let $RR_0 = IER(z_0)$ be the RR for those only exposed to ambient particulate matter pollution; let $RR_1 = IER(z_0 + z_1)$ be the RR for those exposed to both ambient and household sources of particulate matter; and let $RR_{PM}$ be a summary RR of all particulate matter exposure in the given population. We calculate $RR_{PM}$ to be a weighted average of the source specific RRs as follows, $RR_{PM} = p_0 * RR_0 + p_1 * RR_1$.

Let $PAF_0$ and $PAF_1$ be the population attributable fractions for ambient and household sources respectively, and let $PAF_{PM}$ be the PAF for all particulate matter exposure in the population.

A PAF for exposure level $z$ is calculated with the following formula, $PAF_z = \frac{RR_z - RR_{cf}}{RR_z}$. Where $RR_z$ is the RR at exposure level $z$, and $RR_{cf}$ is the RR at the counterfactual level of exposure or TMREL. In this case, $RR_{cf} = IER\left(z_{cf}\right) = 1$, so the equation is simplified to $PAF_z = \frac{RR_z - 1}{RR_z}$.

In the proportional PAF strategy, we calculate one PAF based on the summary RR for the entire population as follows, $PAF_{PM} = \frac{RR_{PM} - 1}{RR_{PM}}$ and proportionally split each component source PAF based on the exposure as follows, $PAF_0 = PAF_{PM} \frac{z_0}{z_{mean}}$ and $PAF_1 = PAF_{PM} \frac{p_1 \times z_1}{z_{mean}}$. As a result, the total particulate matter PAF is equivalent to the sub of all component PAFs, $PAF_0 + PAF_1 = PAF_{PM}$.

# References:


1 van Donkelaar A, Martin R V., Brauer M, *et al.* Global Estimates of Fine Particulate Matter using a Combined Geophysical-Statistical Method with Information from Satellites, Models, and Monitors. *Environ Sci Technol* 2016; **50**: 3762–72.

2 WHO | WHO Global Ambient Air Quality Database (update 2018). *WHO* 2019. https://www.who.int/airpollution/data/cities/en/ (accessed April 3, 2019).

3 Supplementary appendix 1. https://www.thelancet.com/cms/10.1016/S0140-6736(18)32225-6/attachment/997ff617-6a93-45d7-97ab-b45881b9b7c6/mmc1.pdf (accessed April 3, 2019).

4 Shaddick G, Thomas ML, Jobling A, *et al.* Data Integration Model for Air Quality: A Hierarchical Approach to the Global Estimation of Exposures to Ambient Air Pollution. 2016; published online Sept 1. http://arxiv.org/abs/1609.00141 (accessed April 3, 2019).

5 Shaddick G, Thomas ML, Green A, *et al.* Data integration model for air quality: a hierarchical approach to the global estimation of exposures to ambient air pollution. *J R Stat Soc Ser C (Applied Stat* 2018; **67**: 231–53.

6 WHO | WHO Household energy database. *WHO* 2017. https://www.who.int/airpollution/data/household-energy-database/en/ (accessed April 3, 2019).

7 WHO | Global database of household air pollution measurements. *WHO* 2018.

8 Shupler M, Godwin W, Frostad J, Gustafson P, Arku RE, Brauer M. Global estimation of exposure to fine particulate matter (PM2.5) from household air pollution. *Environ Int* 2018; **120**: 354–63.

9 Pope CA, Burnett RT, Krewski D, *et al.* Cardiovascular Mortality and Exposure to Airborne Fine Particulate Matter and Cigarette Smoke. *Circulation* 2009; **120**: 941–8.

10 Semple S, Apsley A, Ibrahim TA, Turner SW, Cherrie JW. Fine particulate matter concentrations in smoking households: just how much secondhand smoke do you breathe in if you live with a smoker who smokes indoors? *Tob Control* 2015; **24**: e205–11.

## Tables

***Table S1: Studies included in metaregression***

| Reference | Source | Time period | ISO | Sex* | Study design | Study size | Minimum exposure (µg/m³) | Maximum exposure (µg/m³) | RR⁺ increase over study exposure range | RR⁺ increase per 10 µg/m3 |
|---|---|---|---|---|---|---|---|---|---|---|
| To et al.[1] | AAP | 1980-2013 | CAN | F | Cohort | 29,549 | 8.52 | 16.42 | 1.22 (1.10-1.34) | 1.28 (1.13-1.45) |
| Turner et al.[2]. | AAP | 1982-2004 | USA | B | Cohort | 669,046 | 8.20 | 17.10 | 1.11 (1.02-1.23) | 1.13 (1.02-1.26) |
| Puett et al.[3] | AAP | 1989-2002 | USA | F | Cohort | 74,412 | 13.20 | 23.40 | 1.05 (0.85-1.25) | 1.05 (0.86-1.24) |
| | | | | M | Cohort | 15,048 | 13.06 | 21.94 | 1.16 (0.83-1.61) | 1.18 (0.81-1.71) |
| Yin et al.[4] | AAP | 1990-2005 | CHN | M | Cohort | 189,793 | 15.50 | 77.10 | 1.14 (0.73-1.78) | 1.02 (0.95-1.10) |
| Burnett, R.[5] | AAP | 1991-2011 | CAN | B | Cohort | 2,256,975 | 4.19 | 11.97 | 1.34 (1.20-1.51) | 1.46 (1.26-1.69) |
| | | | | | Cohort | 2,415,645 | 4.19 | 12.87 | 1.42 (1.29-1.55) | 1.49 (1.34-1.66) |
| | | | | | Cohort | 1,918,570 | 4.19 | 15.30 | 1.11 (1.02-1.21) | 1.10 (1.02-1.19) |
| Hansen et al.[6] | AAP | 1993-2013 | DNK | F | Cohort | 24,172 | 13.49 | 22.71 | 1.35 (1.04-1.77) | 1.39 (1.04-1.86) |
| Coogan et al.[7] | AAP | 1994-2002 | CAN | F | Cohort | 43,003 | 10.12 | 17.68 | 0.97 (0.76-1.25) | 0.97 (0.70-1.35) |
| Clark et al.[8] | AAP | 1995-2011 | USA | B | Cohort | 380,738 | 4.19 | 4.85 | 1.03 (1.01-1.05) | 1.52 (1.15-1.99) |
| Chen et al.[9] | AAP | 1996-2010 | CAN | B | Cohort | 60,076 | 5.94 | 15.26 | 1.10 (1.02-1.19) | 1.11 (1.02-1.21) |
| Wong et al.[10] | AAP | 1998-2011 | CHN, HKG | B | Cohort | 53,905 | 31.90 | 39.70 | 1.41 (1.13-1.72) | 1.55 (1.16-2.01) |
| Park et al.[11] | AAP | 2000-2012 | USA | B | Cohort | 5,135 | 12.09 | 21.31 | 1.20 (0.59-2.40) | 1.22 (0.56-2.59) |
| Weinmayr et al.[12] | AAP | 2000-2008 | DEU | B | Cohort | 3,607 | 14.28 | 19.12 | 1.18 (0.78-1.71) | 1.40 (0.60-3.04) |
| Bowe et al.[13] | AAP | 2003-2012 | USA | B | Cohort | 1,729,108 | 7.54 | 16.06 | 1.13 (1.07-1.18) | 1.15 (1.08-1.22) |

| | | | | | | | | | | |
|---|---|---|---|---|---|---|---|---|---|---|
| Kim et al.[14] | HAP | 1996-2009 | CHN | F | Cohort | 74,941 | 59.45 | 104.26 | 1.37 (0.83-2.26) | 1.07 (0.96-1.20) |
| Hystadt | HAP | 2002-2015 | G | B | Cohort | | | | 0.93 (0.83-1.03) | |
| Zhang et al.[15] | SHS | 1982-2006 | USA | F | Cohort | | 4.19 | 28.74 | 1.16 (1.00-1.35) | 1.06230 (1.00000-1.12999) |
| Houston et al.[16] | SHS | 1985-2001 | USA | B | Cohort | | 4.19 | 24.49 | 1.35 (1.06-1.71) | 1.15927 (1.02911-1.30239) |
| Hayashino et al.[17] | SHS | 1999-2004 | JPN | B | Cohort | | 4.19 | 41.35 | 1.81 (1.06-1.71) | 1.17309 (1.01580-1.15529) |
| Kowall et al.[18]. | SHS | 1999-2001 | DEU | B | Cohort | | 4.19 | 38.19 | 2.50 (1.10-5.60) | 1.30931 (1.02843-1.65981) |
| Ko et al.[19]. | SHS | 2001-2002 | KOR | B | Cohort | | 4.19 | 43.74 | 1.41 (1.10-1.70) | 1.09075 (1.02439-1.14357) |

[*] *M=Male, F=Female, and B=Both.* [+] RR=Relative Risk. Here we also include odds ratios for case-control studies, AAP=Ambient air pollution, HAP=Household air pollution, SHS=Second hand smoke

*Table S2: Overview of bias covariates adjusted for in meta-regressions for each study*

| Reference | Subpop-ulation [a] | Pop-ulation exposure [b] | Self-reported exposure [c] | Multiple exposure measure ments [d] | Self-reported outcome [e] | Unblinded outcome [f] | Reverse causation [g] | Randomi zation [h] | Statistical control for counfound-ing | Follow-up |
|---|---|---|---|---|---|---|---|---|---|---|
| To et al.[1] | 0 | 1 | 0 | 1 | 0 | 0 | 0 | 1 | 0 | 1 |
| Turner et al.[2]. | 0 | 0 | 0 | 1 | 0 | 0 | 0 | 1 | 0 | 0 |
| Puett et al.[3] | 0 | 0 | 0 | 0 | 1 | 0 | 0 | 1 | 1 | 1 |
| Yin et al.[4] | 0 | 0 | 0 | 1 | 0 | 0 | 0 | 1 | 1 | 2 |
| Burnett, R.[5] | 0 | 0 | 0 | 0 | 0 | 0 | 0 | 1 | 2 | 2 |
| | 0 | 0 | 0 | 0 | 0 | 0 | 0 | 1 | 2 | 2 |
| | 0 | 0 | 0 | 0 | 0 | 0 | 0 | 1 | 2 | 2 |
| Hansen et al.[6] | 0 | 0 | 0 | 0 | 0 | 0 | 0 | 1 | 1 | 1 |
| Coogan et al.[7] | 0 | 0 | 0 | 1 | 1 | 0 | 0 | 1 | 2 | 2 |
| Clark et al.[8] | 0 | 0 | 0 | 1 | 0 | 0 | 0 | 1 | 2 | 2 |
| Chen et al.[9] | 0 | 1 | 0 | 1 | 0 | 0 | 0 | 1 | 0 | 2 |
| Wong et al.[10] | 0 | 0 | 0 | 0 | 0 | 0 | 0 | 1 | 0 | 1 |
| Park et al.[11] | 0 | 0 | 0 | 1 | 0 | 0 | 0 | 1 | 0 | 0 |
| Weinmayr et al.[12] | 0 | 0 | 0 | 1 | 0 | 0 | 0 | 1 | 1 | 1 |
| Bowe et al.[13] | 0 | 1 | 0 | 1 | 0 | 0 | 0 | 1 | 1 | 1 |
| Lim et al. | 0 | 0 | 0 | 1 | 0 | 0 | 0 | 1 | 0 | 0 |
| Kim et al.[14] | 0 | 0 | 1 | 1 | 0 | 1 | 0 | 1 | 0 | 0 |

| | | | | | | | | | | |
|---|---|---|---|---|---|---|---|---|---|---|
| Hystad et al. | 0 | 0 | 1 | 1 | 0 | 0 | 0 | 1 | 0 | 2 |
| Zhang et al.[15] | 0 | 0 | 1 | 0 | 1 | 0 | 0 | 1 | 1 | 2 |
| Houston et al.[16] | 0 | 0 | 0 | 0 | 0 | 0 | 0 | 1 | 0 | 2 |
| Hayashino et al.[17] | 0 | 0 | 1 | 1 | 0 | 0 | 0 | 1 | 1 | 2 |
| Kowall et al.[18]. | 0 | 0 | 1 | 0 | 1 | 0 | 0 | 1 | 1 | 2 |
| Ko et al.[19]. | 0 | 0 | 1 | 0 | 1 | 1 | 0 | 1 | 1 | 2 |

[a] **Subpopulation:** Study assessed subpopulation (vs. overall population) *(0=no; 1=yes)*

[b] **Population exposure:** Exposure was assessed on population level (vs. individual level) *(0=no; 1=yes)*

[c] **Self-reported exposure:** Exposure was self-reported *(0=no, 1=yes)*

[d] **Multiple exposure measurements:** Exposure was measured multiple times over the course of the study, (vs. just at baseline) *(0=no; 1=yes)*

[e] **Self-reported outcome:** outcome self-reported *(0=no, 1=yes)*

[f] **Unblinded outcome:** assessment of outcome is blind to exposure *(0=blinded, 1=unblinded)*

[g] **Reverse causation:** Risk of reverse causation *(0=minimal or no risk of reverse causation, 1=risk of reverse causation).*

[h] **Randomization:** A randomization variable: *(0= randomized study, 1= non-randomized study)*

[I] **Statistical control for confounding** (0*=randomization or outcome controlled for all major known confounders and age, sex, education, income, and all critical determinants of outcome, 1= controlled for age, sex, and other critical determinants, 2=controlled for only age and sex)*

[j] **Follow-up** degree to which study subjects were lost to follow-up *(0=greater than 95% follow-up, 1= follow up of 85-95%, 2=less than 85% follow up)*

***References***


1 To T, Zhu J, Villeneuve PJ, *et al.* Chronic disease prevalence in women and air pollution — A 30-year longitudinal cohort study. *Environment International* 2015; **80**: 26–32.

2 Turner MC, Jerrett M, Iii CAP, *et al.* Long-Term Ozone Exposure and Mortality in a Large Prospective Study. 2016; **193**: 1134–42.

3 Puett RC, Hart JE, Schwartz J, Hu FB, Liese AD, Laden F. Are Particulate Matter Exposures Associated with Risk of Type 2 Diabetes? 2011; **384**: 384–9.

4 Yin P, Brauer M, Cohen A, *et al.* Long-term Fine Particulate Matter Exposure and Nonaccidental and Cause-specific Mortality in a Large National Cohort of Chinese Men. 2017; : 1–11.

5 Burnett R. Cox Proportional Survival Model Hazard Ratios from Census Year to 2011 for Adults Aged 25 to 89 in CanCHEC Cohort. .

6 Hansen AB, Ravnskjær L, Loft S, *et al.* Long-term exposure to fine particulate matter and incidence of diabetes in the Danish Nurse Cohort. *Environment International* 2016; **91**: 243–50.

7 Coogan PF, White LF, Yu J, *et al.* PM2.5 and Diabetes and Hypertension Incidence in the Black Women's Health Study. *Epidemiology* 2015; **27**: 1.

8 Clark C, Sbihi H, Tamburic L, Brauer M, Frank LD, Davies HW. Association of Long-Term Exposure to Transportation Noise and Traffic-Related Air Pollution with the Incidence of Diabetes : A Prospective Cohort Study. 2016; : 1–10.

9 Chen H, Burnett RT, Kwong JC, *et al.* Risk of incident diabetes in relation to long-term exposure to fine particulate matter in Ontario, Canada. *Environmental Health Perspectives* 2013; **121**: 804–10.

10 Wong CM, Lai HK, Tsang H, *et al.* Satellite-Based Estimates of Long-Term Exposure to Fine Particles and Association with Mortality in Elderly Hong Kong Residents. 2015; **123**: 1167–72.

11 Park SK, Adar SD, Neill MSO, *et al.* Original Contribution Long-Term Exposure to Air Pollution and Type 2 Diabetes Mellitus in a Multiethnic Cohort. 2015; **181**: 327–36.

12 Weinmayr G, Hennig F, Fuks K, *et al.* Long-term exposure to fine particulate matter and incidence of type 2 diabetes mellitus in a cohort study : effects of total and traffic-specific air pollution. *Environmental Health* 2015; : 1–8.

13 Bowe B, Xie Y, Li T, Yan Y, Xian H, Al-aly Z. Articles The 2016 global and national burden of diabetes mellitus attributable to PM 2 · 5 air pollution. *The Lancet Planetary Health* 2018; **2**: e301–12.

14 Kim C, Seow WJ, Shu X, Bassig BA, Rothman N, Chen BE. Cooking Coal Use and All-Cause and Cause-Specific Mortality in a Prospective Cohort Study of Women in Shanghai , China. 2016; : 1384–9.

15 Zhang L, Curhan GC, Hu FB, Rimm EB, Forman JP. Association Between Passive and Active Smoking and Incident Type 2 Diabetes in Women. *Diabetes Care* 2011; **34**: 892–7.

16 Houston TK, Person SD, Pletcher MJ, Liu K, Iribarren C, Kiefe CI. Active and passive smoking and development of glucose intolerance among young adults in a prospective cohort: CARDIA study. *BMJ* 2006; **332**: 1064–9.

17 Hayashino Y, Fukuhara S, Okamura T, *et al.* A Prospective Study of Passive Smoking and Risk of Diabetes in a Cohort of Workers The High-Risk and Population Strategy for Occupational Health Promotion (HIPOP-OHP) study. 2008. DOI:10.2337/dc07-1905.

18 Kowall B, Rathmann W, Strassburger K, *et al.* Association of passive and active smoking with incident type 2 diabetes mellitus in the elderly population: the KORA S4/F4 cohort study. *European Journal of Epidemiology* 2010; **25**: 393–402.

19 Ko K-P, Min H, Ahn Y, *et al.* A Prospective Study Investigating the Association Between Environmental Tobacco Smoke Exposure and the Incidence of Type 2 Diabetes in Never Smokers. *Annals of Epidemiology* 2011; **21**: 42–7.
